# HPV-associated anal lesions in HIV+ patients: long-term results regarding quality of life

**DOI:** 10.1007/s00384-020-03567-1

**Published:** 2020-03-26

**Authors:** Paul Wesselmann, Carolynne Schwarze-Zander, Christoph Boesecke, Jürgen Rockstroh, B. Stoffels, Tim O. Vilz, Tim R. Glowka, J. C. Kalff, Martin W. von Websky

**Affiliations:** 1 Department of Surgery, University Hospital of Bonn, Sigmund-Freud-Str. 25, Bonn, Germany; 2 Department of Medicine I, University Hospital of Bonn, Sigmund-Freud-Str. 25, Bonn, Germany

**Keywords:** Anal intraepithelial neoplasia, HIV, Quality of life, HPV, Condyloma, SF-36

## Abstract

**Purpose:**

HIV infection and concomitant HPV-associated anal lesions may significantly impact on patients’ quality of life (QoL), as they are predicted to have negative effects on health, psyche, and sexuality.

**Material and methods:**

Fifty-two HIV+ patients with HPV-associated anal lesions were enrolled in a survey approach after undergoing routine proctologic assessment and therapy for HPV-associated anal lesions if indicated over a time span of 11 years (11/2004–11/2015). Therapy consisted of surgical ablation and topic treatment. QoL was analyzed using the SF-36 and the CECA questionnaires.

**Results:**

Fifty-two of 67 patients (77.6%) were successfully contacted and 29/52 provided full information. The mean age was 43.8 ± 12.8 years. The median follow-up from treatment to answering of the questionnaire was 34 months. Twenty-one percent (6/29) of the patients reported suffering from recurrence of condyloma acuminata, three patients from anal dysplasia (10.3%). In the SF-36, HIV+ patients did not rate their QoL as significantly different over all items after successful treatment of HPV-associated anal lesions. In the CECA questionnaire, patients with persisting HPV-associated anal lesions reported significantly higher emotional stress levels and disturbance of everyday life compared to patients who had successful treatment (71.9/100 ± 18.7 vs. 40.00/100 ± 27.4, *p* = 0.004). Importantly, the sexuality of patients with anal lesions was significantly impaired (59.8/100 ± 30.8 vs. 27.5/100 ± 12.2, *p* = 0.032).

**Conclusion:**

HPV-associated anal lesions impact significantly negative on QoL in HIV+ patients. Successful treatment of HPV-associated anal lesions in HIV+ patients improved QoL. Specific questionnaires, such as CECA, seem to be more adequate than the SF-36 in this setting.

**Electronic supplementary material:**

The online version of this article (10.1007/s00384-020-03567-1) contains supplementary material, which is available to authorized users.

## Introduction

According to the literature, topical treatment (imiquimod) and surgical ablation are equivalent first-line treatment options for HPV-associated anal lesions. While surgical therapy is associated with shorter treatment duration and better outcome [1] [2], topical treatment with imiquimod is less invasive and more cost-effective.

Physical illnesses in general are not limited to disease-specific symptoms and sequelae, but may also have a psychological impact on the patient. This often goes hand in hand with fear of social isolation and stigmatization, especially in infectious and sexually transmitted diseases. HIV and HPV infections, in particular, can severely limit a patient’s quality of life (QoL) in this aspect [3]. While HIV infection, an incurable systemic disease, and concomitant HPV infection, which can become symptomatic in the form of anogenital warts, precancerous disease, or anal carcinoma, are very different in nature, both diseases, being mainly sexually transmitted, affect sexual function and, specifically, the patient’s health [4].

It is known that HIV infection significantly reduces QoL when compared to the HIV-negative population [4]. The same holds true for HPV-associated anal lesions as shown in 2013 by Dominiak-Felden et al. in their study on vaginal HPV-associated lesions [5].

To date, HIV therapy relies on antiretroviral agents. However, an optimal therapeutic regimen for HPV-associated anal lesions has not been developed. When selecting a therapy approach from the available treatment options for HPV-associated disease, it may be helpful to consider the impact on QoL, which is not only influenced by the illness itself, but also by side effects, duration, and effectiveness of the therapy. Also, adherence to therapy may be affected by the perceived impact on QoL [6].

In the treatment of HIV, quality of life has already been established as a decisive aspect in the selection of therapy management [7]. In general, however, improvement in the quality of life can be observed in all antiviral treatment regimens [8]. Since according to the Swiss Statement of 2008, sexual intercourse among HIV+ and HIV-negative partners under antiretroviral therapy (ART) is no longer considered contagious “in the scene,” the significance of the diagnosis has also positively changed perception of HIV [9]. In this large study, the risk of infection during sexual intercourse in pairs without condom use was seen as very low. The risk of infection under ART is perceived to be lower and may thus have a corresponding effect on sexuality. [10]. However, this potentially reduced risk of HIV infection does not apply to the frequent co-infections (such as HPV) which may in turn become more widespread due to lower compliance with mechanical infection protection (use of condoms) [9].

The different manifestations of HPV infections affect the quality of life to varying degrees. In 2013, Dominiak-Felden et al. found that women with genital warts had a lower quality of life than women with precancerous lesions, which are more dangerous but not as striking, as they are smaller and potentially less noticeable [5]. In 2010, Mortensen and Larsen analyzed the psychological burden of genital condyloma in a qualitative study interviewing a young population. The authors found that the greatest burden for patients is the restriction in their sexual life and the uncertainty of a lasting treatment success, which results in varying effectiveness and duration of treatment [11].

In this follow-up study, QoL after the treatment of HPV-associated anal lesions in HIV+ patients was examined. In addition, we investigated to what extent successful treatment of HPV-associated anal lesions in HIV+ patients improved their quality of life and whether this improvement remained significant when controlled for the main diagnosis of HIV+.

## Methods

**Fig. 1:**
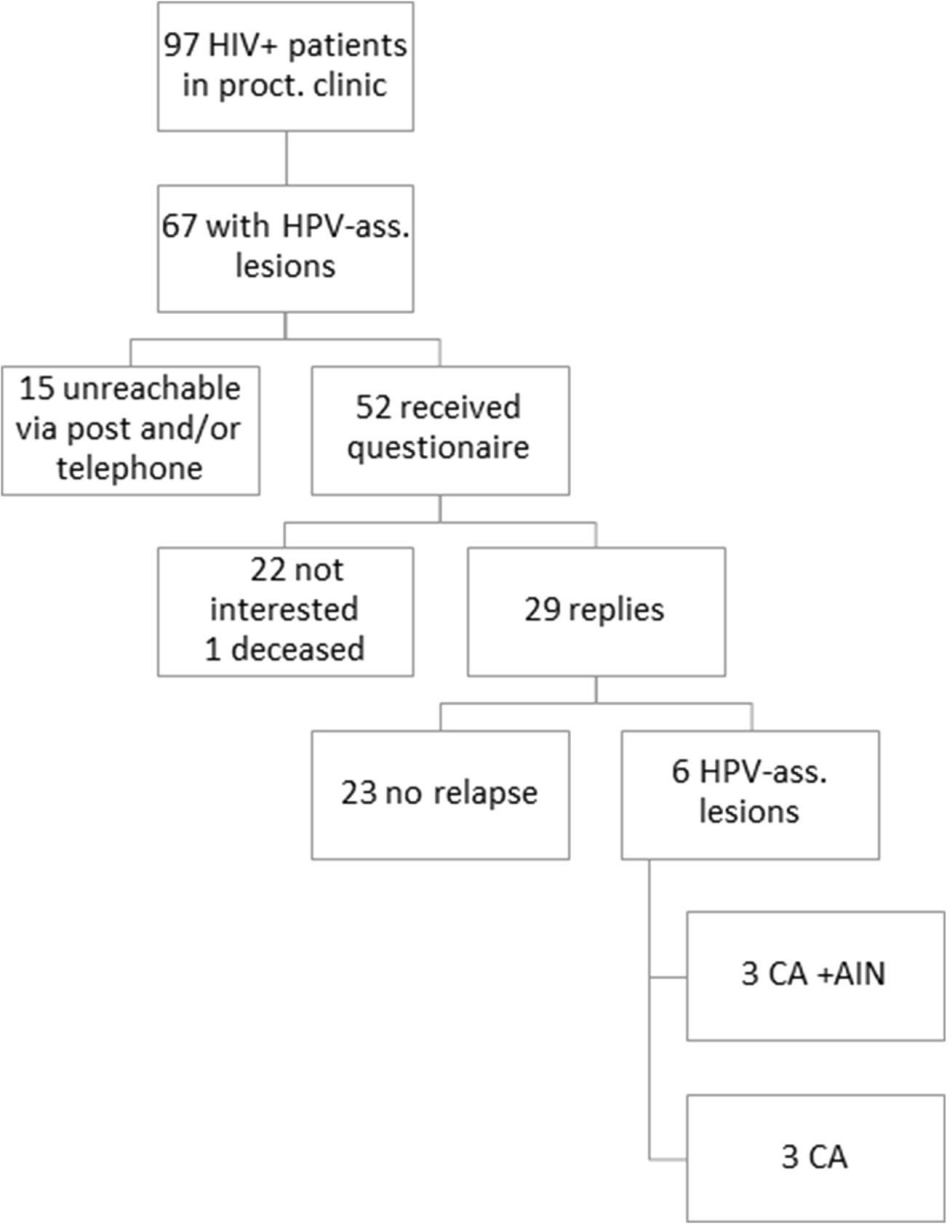
Flowchart of patient recruitment. Sixty-seven of the 97 HIV+ patients who were seen by the proctological outpatient clinic during the study period had HPV-associated anal lesions. Of these, 29 responded by filling in the study questionnaire on QoL. Of these, 23 did not have a relapse, while six patients had new or relapsed HPV-associated anal lesions

Following approval by the University Hospital of Bonn Ethics Committee (ID number 233/16) data from 67 HIV+ patients treated for 10 years (11/2004–11/2015) for HPV-associated lesions such as condyloma acuminata or anal intraepithelial neoplasia (called CA or AIN in the following) was analyzed at our center (Fig. 1).

All 29 patients who provided a valid response were retrospectively characterized according to age, HIV therapy parameters, and HPV status. In detail, viral load, CD4 count, and the CDC classification were recorded. In the CDC classification, clinical symptoms are summarized in the categories A (asymptomatic), B (non-AIDS-defining illnesses), and C (AIDS-defining illnesses). A questionnaire on long-term follow-up (treatment success yes/no) quality of life (SF-36), as well as the CECA questionnaire (which specifically addresses the psychological stress in the setting of the above-mentioned anal lesions), was sent to all patients.

The corresponding answers were entered in a database in an anonymized fashion and statistically evaluated [12, 13].

The survey focused on QoL in the presence of HPV-associated anal lesions such as CA and/or AIN. All patients with a history of CA and/or AIN at least once during their proctological treatment were contacted. The above-mentioned questionnaires on general quality of life (SF-36) and the more specific CECA questionnaire took about 12 min in total to complete. The questionnaires were sent by mail and patients were asked to return the forms in a pre-paid neutral envelope. Patients who initially did not send a reply were contacted twice by telephone every 2 weeks and asked again to participate. The questionnaires also referred to CA or AIN recurrence to enable assessment of the long-term outcome after treatment.

The SF-36 questionnaire is a well-documented and internationally employed instrument for measuring health-related quality of life and has been translated into 40 languages [14]. The results were evaluated according to the handbook “Questionnaire on the general state of health,” which guarantees a cross-study comparison of reliability and validity [15]. With its 36 items, the SF-36 covers the physical, mental, and social dimensions. The different dimensions are divided into eight scales concerning only the subjective health: physical functioning, physical role function, physical pain, general health perception, vitality, social functioning, emotional role function ability, and psychological well-being.

The second and more specific of the questionnaires employed in this study, the CECA questionnaire, concentrates on the psychological impact of anal lesions and sexual health. CECA is a Spanish acronym for “Specific Questionnaire on Condylomata Acuminata” and it assesses general fears of infection and its consequences, its influence on mood in everyday life, and its consequences on sexual function in terms of quality and quantity [12].

The CECA questionnaire was developed by Badia et al. in 2005 and has been translated into English, Chinese, and German [16, 17]. The questions of the CECA questionnaire are similar to the DQLI (Dermatology Life Quality Index), but include also questions on morbidity related to HPV-associated anogenital lesions [18]. In our study, the CECA questionnaire was also adopted for patients with AIN, since these patients are often associated with CA. The CECA questionnaire consists of six questions about emotionality and four questions about sexuality.

In the evaluation of the survey, natural numbers were assigned to the answer options “always,” “mostly,” “sometimes,” “rare,” and “never”; the higher the number, the “better” the patients rated themselves in the individual question. In order to facilitate comparison of the individual question dimensions, they were combined and converted to a score between 0 and 100. A score of 0 describes the worst quality of life that can be achieved and a score of 100 the best. Three groups of patients were compared (Table 1). For statistical evaluation, the two groups “with CA” and “CA and AIN” were combined due to small sample size. This was compared to the sample of patients without recurrent HPV-associated anal lesions.

**Table 1:** Epidemiology, immunologic status, and therapy

	Total (*n* = 29)	Group I (HIV+ with successfully treated HPV lesion, *n* = 23))	Group II (HIV+ with HPV lesion, *n* = 6)
Age (years); *p* = 0.18			
Mean	43.8	45.4	37.5
Min	18.8	18.8	25.5
Max	61.9	61.9	60.6
SD	12.8	12.4	13.4
CD4 (cells/μl); *p* = 0.437			
Mean	517	535.6	451
Min	156	156	215
Max	1064	1064	648
SD	229	245	161
Viral load (cp/μl); *p* = 0.629			
Mean	160,691	195,955	25,513
Min	0	0	0
Max	4,029,233	4,029,233	153,018
SD	749,671	841,494	62,464
CDC category			
A	14	11	3
B	4	2	2
C	5	5	0
No data	6	5	1
Follow-up			
No complains	18	16	2
CA	6	5	2
Dysplasia	7	4	2
Therapy			
Imiquimod	5	3	2
Surgery	12	10	2

### Group overview

#### Group I

HIV+ patients after successful treatment of CA or AIN (*n* = 23).

#### Group II

HIV+ patients with current CA or AIN detection (*n* = 6).

#### Group III (reference group)

Reference values of the normal population, men between 40 and 49 years, based on the Federal German Health Survey, Bellach and Ellert 1998 (only applied to the SF-36 questionnaire)

### Data evaluation and statistics

The statistical analyses were performed with SPSS version 23.0 for Windows (SPSS, Chicago, IL, USA). Mean values and minima and maxima as well as standard deviations are reported for descriptive statistics regarding quantitative characteristics. Absolute and relative frequencies (in %) are specified for qualitative characteristics.

For categorical variables, we used the chi-square test. We compared continuous variables with the Mann-Whitney *U* test or Student’s *t* test in the presence of a normal distribution which was first tested using the Kolmogorov Smirnov test. *P* values < 0.05 were considered statistically significant.

To compare the screened group of patients and the reference population, we used the calculation of a “*z*-value,” which is a measure for standard deviation. Values greater than 1.96 or less than − 1.96 correspond to a confidence interval greater than 95%. The comparison of the three groups is therefore performed with *z*-value calculation and *t* test.

## Results

### Quality of life, sexual function, and psychological stress in HIV+ patients with HPV-associated anal lesions

#### Patient characteristics and reply rates

Of the 97 HIV+ patients who presented at the proctological outpatient department of the University Hospital Bonn in the period under study, 67 patients (69.0%) had at least one HPV-associated lesion, either CA or AIN. Of these 67 patients, 52 were successfully contacted and asked to participate in the mail survey that included the SF-36 questionnaire and the CECA questionnaire. Of these 52 patients, 29 (55.7%) fully answered the survey (see also Fig. 1). In the 29 valid responses, almost 80% of patients stated that they were free of symptoms after successful therapy (surgical or topical) (23/29; 79.3%). The remaining six patients reported recurrence of CA (6/29; 20.7%), and three patients also reported recurrence of anal intraepithelial dysplasia (3/29; 10.3%). The mean patient age was 43.8 ± 12.8 years. Patients with recurrent CA or AIN displayed a tendency to be younger: 37.5 ± 13.4 years vs. 45.4 ± 12.4 years, there was no significance, *t* test (*p* = 0.18). Clinical follow-up data was available from 29 patients, the median follow-up time was 34 months (IQR = 15; 59), ranging from 1 to 9 years postoperatively.

In the CDC classification, half of the patients in each group could be classified in category A (Table 1).

Of the patients with current HPV-associated lesion, two had been inconspicuous at the last control examination and thus showed a recurrence or reinfection.

##### Quality of life with HPV-associated lesions (SF-36 questionnaire)

The evaluation of the SF-36 subscales revealed no significant difference in the QoL items between the groups with and without current or recurrent HPV-associated anal lesions.

Furthermore, the QoL of our study collective did not differ significantly from the reference values of a HIV-negative male normal population aged 40–49 (reference values taken from Bellach et al. 2000). The results of the comparisons between groups I and II and the reference population group III are given in Table 2.

**Table 2:** QoL as assessed by the SF-36 (scale evaluation)

SF-36 scale level 0–100	Group I (HIV+ with successfully treated lesion) mean value ± SD	Group II (HIV+ with HPV lesion) mean value ± SD	Group III (reference population) mean value ± SD	Group I vs. II: difference (*p* value)	Group I vs. III: difference (*z*-value)	Group II vs. III: difference (*z*-value)
Physical functioning	82.4 ± 23.9	75.0 ± 26.5	91.32 ± 13.43	− 7.4 (0.514)	− 8.9 (−0.663)	− 16.3 (−1.214)
Role limitations due to physical health	66.3 ± 41.7	54.2 ± 45.9	88.42 ± 25.68	− 12.1 (0.539)	− 22.1 (−0.861)	− 34.3 (−1.336)
Pain	82.9 ± 21.3	68.8 ± 27.6	71.33 ± 25.01	− 14.1 (0.185)	11.6 (−0.464)	− 2.5 (−0.1)
General health	63.7 ± 16.6	50.5 ± 11.5	68.13 ± 16.08	− 13.2 (0.800)	− 4.5 (−0.280)	− 17.6 (−1.094)
Vitality	55.1 ± 18.8	44.2 ± 24.2	64.20 ± 16.26	− 10.9 (0.240)	− 9.1 (−0.560)	− 20.0 (−1.23)
Social functioning	76.1 ± 25.3	62.5 ± 27.4	89.20 ± 17.40	− 13.6 (0.258)	− 13.1 (−0.753)	− 26.7 (−1.534)
Role limitations due to emotional problems	66.7 ± 47.1	55.6 ± 45.5	91.93 ± 23.60	− 11.1 (0.611)	− 25.3 (−1.072)	− 36.4 (−1.542)
Emotional well-being	68.3 ± 17.4	55.3 ± 12.8	75.23 ± 14.83	− 12.9 (0.100)	− 6.9 (−0.465)	− 19.9 (−1.341)
Physical summary score	50.6	45.6	NA*	− 5.1 (0.222)	NA	
Mental summary score	45.6	39.3	NA	− 6.3 (0.174)	NA	

Summary scores not calculated for reference population

##### Sexual function and psychological stress in HIV+ patients (CECA questionnaire)

Patients without HPV-associated lesions stated overall that they were less affected by emotional and sexual stress. The option “never” was chosen most frequently in 4/6 of the questions; these were the disease-specific questions (1, 2, 3, and 6). Patients with HPV-associated lesions were “often” to “sometimes” concerned about their infection (Table 3).

**Table 3:** QoL as assessed by the CECA questionnaire

Question	Group I (HIV+ with successfully treated HPV lesion)	Group II (HIV+ with HPV lesion)	*p* value
1. I am afraid that the lesions will not disappear.	3.96 ± 1.147	2.40 ± 1.342	*0.007*
2. I am anxious to know whether I am going to recover from the infection for good.	4.00 ± 1.000	2.40 ± 1.342	*0.004*
3. I worry about whether the warts will get worse or if there will be some complications	3.83 ± 1.072	2.67 ± 1.366	*0.031*
4. My state of mind is upset (anxiety, depression, sadness, uneasiness…).	3.74 ± 0.915	2.83 ± 0.753	*0.041*
5. I feel more insecure.	3.65 ± 0.775	2.00 ± 1.00	*0.000*
6. Knowing that I have the illness affects me in my daily life.	3.09 ± 0.996	3.33 ± 1.506	0.157
7. My sexual drive has decreased.	3.45 ± 1.262	2.00 ± 0.632	0.15
8. I feel worried during the act.	3.64 ± 1.293	2.00 ± 0.707	*0.012*
9. I avoid sexual relations.	3.50 ± 1.472	2.50 ± 0.548	0.096
10. My sexual relations have decreased in quality and/or frequency.	2.91 ± 1.630	1.67 ± 0.816	0.094

Significant *p* values are shown in italics

##### Items for sexual function

The sexual items in this questionnaire consisted of questions concerning sexual pleasure, care during sexual intercourse, avoidance of sexual contacts, and loss of sexual contacts. The patients specified that they had more concerns and suffered more if they still had HPV-associated anal lesions.

##### Overview of the dimensions surveyed in the CECA questionnaire

Patients with recurrent HPV-associated anal lesions reported that the disease had a significant psychological impact on their daily lives compared to patients without anal lesions. Mean value of the emotional dimension of group I was 71.9 ± 18.7 vs. 40.00 ± 27.4 group II, *t* test (*p* = 0.04). This means that the groups differ by 30 out of 100 points.

**Fig. 2:**
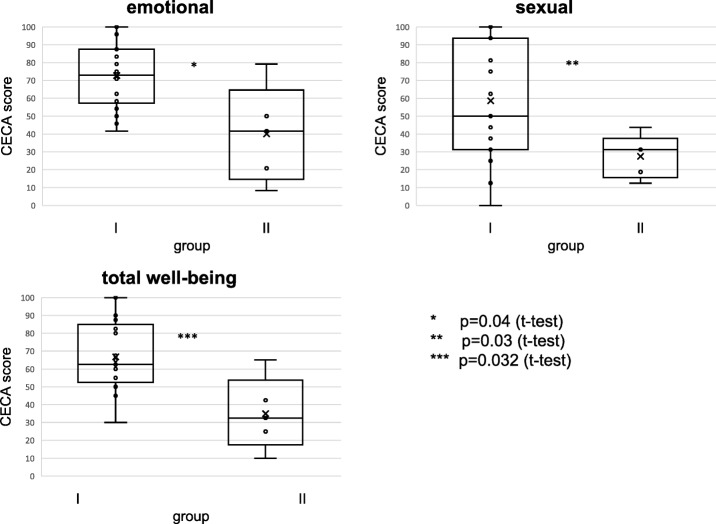
Overview of QoL as assessed by the CECA questionnaire. Comparison of group I with successfully treated and of group II with recurrent HPV-associated anal lesions shows a significant difference. In the emotional, sexual, and total well-being dimension, the patients with successfully treated lesions display a significantly higher score, which translates to significantly better QoL

There was a large difference in quality of life regarding the sexual dimension: 59.8 ± 30.8 in group I vs. 27.5 ± 12.2 in group II, *t* test results (*p* = 0.03). Also in the overall QoL, the two groups differed significantly: group I 66.1 ± 18.7 vs. 35.00 ± 20.54 group II, *t* test (*p* = 0.032), meaning that a successful anal HPV lesion treatment improves quality of life (Fig. 2).

## Discussion

In this study, we show that considerable improvement of quality of life can be achieved through successful treatment of HPV-associated anal lesions in an HIV+ cohort. We used two psychometric tools to assess QoL, the SF-36 and the CECA questionnaire, and we documented a better sensitivity for the CECA tool in this setting.

We successfully contacted 52 patients, of whom, however, 22 were unwilling to partake in this study due to a high level of frustration experienced in the course of their personal medical history.

The SF-36 survey revealed neither a significant reduction of the subjective well-being of the HIV+ collective compared to the normal population, nor an improvement of the QoL as measured by SF-36 after successful treatment of the HPV-associated lesions. These results contradict the findings of Schofield et al. who described the negative influence of HIV and HPV on quality of life [3]. In our cohort, the results of the SF-36 questionnaire showed no significant differences in quality of life across all items. Possibly, our number of patients was insufficient to reach significant results in the SF-36. However, the CECA questionnaire was able to detect such differences in our study population. The CECA questionnaire therefore seems to be more adequate than the SF-36 in this setting and should be considered a useful tool to measure QoL in in HIV+ patients with HPV-associated anal lesions. In order to deal more specifically with the emotional strain and the restrictions on sexuality experienced by the patients, further data were collected with the CECA questionnaire as a long-term follow-up in patients after therapy for CA or AIN. First, it can be noticed that patients with successfully treated lesions achieved significantly better values with a difference of 30 out of 100 points on the emotional scale. To date, no such survey has been conducted in a similar collective. However, Mortensen and Larsen had similar results gained through interviews. In their analysis, patients described in interviews that their greatest burden was the uncertainty about success, duration, and effectiveness of their treatment [11]. The authors thus identified the need for comprehensive consultation and education of the patients, with a focus on the possible long and difficult course of the disease [11]. While HIV infection, a potentially life-threatening disease, reduces the quality of life, a combined infection of HIV and HPV reduces the quality of life considerably more [4]. In fact, we were unable to detect any difference in QoL in our HIV+ population compared to the general population. Mortensen and Larsen’s call for comprehensive counseling and education in order to prepare patients for an extended course of the disease must therefore play a central role in the care of HIV+ patients with HPV-associated lesions. In the CECA questionnaire, the sexuality dimension was investigated in more detail. We found that a patient’s sexuality is especially impaired in the presence of HPV-associated anal lesions. The questions on sexuality had the poorest values in patients with unsuccessfully treated HPV-associated lesions. The patients without HPV-associated declared a significantly better sexual life (59.8 ± 30.8 vs. 27.5 ± 12.2, *p* = 0.032). This further indicates that visible infectious diseases render patients more insecure, as already been reported by Dominiak-Felden et al. in 2013 [5]. Also, in the interviews carried out by Mortensen and Larsen, restrictions on sexual life represented a central burden for the patients [11]. Therefore, it can be summarized that limitations of sexuality in these patients should also be openly addressed in educational discussions.

Overall, the long-term follow-up with 23/29 (79%) treatment-free patients in remission and six (21%) patients with recurrences was satisfactory and clearly better compared to remission rates in other studies. In their follow-up study, Nadal et al. had a complete remission rate of 46% after topical treatment [19]. Surgical treatment of anal condylomata or dysplasia seems to have a better outcome after 4 weeks compared to topical treatment with imiquimod, as we were able to show in a comparative study in 2018 [1]. When HPV-associated anal lesions have a considerable influence on the QoL, as we demonstrate here in HIV+ patients, a short and effective therapy is called for.

As a limitation of our study, the small number of six patients with recurrent disease renders it difficult to infer other conclusions from this group in comparison to the general HIV+ population with HPV-associated anal lesions. Any possibility of enlarging the sample remains difficult, as this disease combination is rare and the willingness to participate in a survey on quality of life and sexuality is generally low. We also experienced this in the > 20 patients who were unwilling to participate. It could be speculated that the 22 patients who refused to participate in the study at time of contact had no current clinical problems and therefore had no interest in participating in this study. Clearly, a multicenter study with a larger collective would be optimal to confirm our results. Furthermore, the classification of the patients into the groups with successful treatment and with recurrent HPV-associated anal lesions was done according to self-reported status. However, self-assessment is obviously associated with a methodological uncertainty. In the telephone conversation with the patients, some indicated that they did not know about their current CA/AIN status. Ideally, a clinical follow-up is recommended.

Nevertheless, the results of our survey on QoL in HPV-associated lesions underline their importance and their serious impact on quality of life. Therefore, HPV-associated anal lesions should be screened for regularly in HIV+ patients, considered in the overall therapy and treated early.

Finally, the value of HPV vaccination must be highlighted for prevention of these lesions, as it has the potential to prevent anal, cervical, penile, and oral HPV lesions in men and women [20, 21]. Successful treatment of HPV-associated lesions in HIV+ patients significantly improved QoL concerning overall physical health as well as emotional and sexual health parameters. Due to the impact on QoL and the potentially underestimated recurrence of HPV-associated anal lesions, proctologic follow-up and treatment should be given high priority in the long-term care of HIV+ patients.

## Electronic supplementary material

ESM 1 (XLSX 15 kb).
